# The Moral Development of the Child: An Integrated Model

**DOI:** 10.3389/fpubh.2013.00057

**Published:** 2013-11-18

**Authors:** Hing Keung Ma

**Affiliations:** ^1^Department of Education Study, Centre for Child Development, Hong Kong Baptist University, Kowloon Tong, Hong Kong, China

**Keywords:** moral development, psychological needs, altruism, human relationships, justice reasoning

## Abstract

Previous theories of moral development such as those by Piaget and Kohlberg usually focused on the cognitive or rational aspect, and seldom included the affective aspect in their construction. The characteristics of the stages of moral development in the present paper are elaborated with special reference to psychological needs, altruism and human relationships, and justice reasoning. The three stages are: (1) Physical Survival, Selfishness, and Obedience, (2) Love Needs, Reciprocal Altruism, and Instrumental Purpose; and (3) Belongingness Needs, Primary Group Altruism, and Mutual Interpersonal Expectations. At Stage 1, a deep and profound attachment to parents, empathy toward the significant others, and obedience to authorities all contribute to the physical survival of a person at this stage. People at Stage 2 are self-protective, dominant, exploitative, and opportunistic. The need to love and to be loved is gratified on the basis of reciprocal altruism. People at Stage 3 have a strong desire to gratify their belongingness needs to a primary group. They are willing to sacrifice for the benefits of the group at great cost. While the psychological needs and altruism are related to the affective aspect of moral development, the justice reasoning is related to the cognitive aspect. The proposed theoretical model attempts to integrate the affective and cognitive aspects of moral development, and prototypic responses to questions related to hypothetical moral dilemmas are presented to substantiate the proposed stage structures. It is hypothesized that the sequence of these three stages is invariant of person and culture.

This paper attempts to establish the stages of moral development with special reference to psychological needs, altruism and human relationships, and justice reasoning.

A number of theories of moral development and behavior have been established in the past few decades (1–6). These theories can roughly be divided into the following four categories: (1) feeling or emotion aspect: these theories emphasize the affective aspect of moral development and include a number of altruism theories. Examples include Aronfreed’s (7) theory of altruistic and sympathetic behavior, Eisenberg’s (8, 9) theory of pro-social development and behavior, and Hoffman’s (10) theory of empathy and moral development. (2) Behavioral aspect: these theories mainly deal with moral behavior. Examples include Eysenck’s (11) behavioral theory of morality and Mischel and Mischel’s (12) cognitive social learning theory of morality. (3) Cognitive aspect: these theories focus on moral judgment and moral reasoning. Examples include Piaget’s (13) theory of moral judgment and Kohlberg’s (14, 15) theory of moral development. (4) Integrated perspectives: a number of theorists have also attempted to propose theories which integrate two or three of the affective, behavioral, and cognitive aspects of morality. Examples include Bandura’s (16) social cognitive theory of moral thought and action, Blasi’s (17) integration of moral understanding and moral personality, Gibbs’ (18) integration of Kohlberg’s and Hoffman’s theories of morality, Krebs and Van Hesteren’s (19) integrative model of altruism development, and Rest’s (20) four components model of morality. There are also many other important and useful theories, e.g., Damon’s (21) theory of development of positive justice, Enright’s (22) theory of moral development of forgiveness, Gilligan’s (23) theory of care and responsibility, Loevinger’s (24) theory of ego development, Haan’s (25, 26) social constructivist perspective on practical morality, and Hogan’s (27) theory of personality and moral development. Another important theory is concerned the moral identity by Aquino and his colleagues (28, 29), Blasi (30), and Lapsley and Narvaez (31).

The use of fMRI (Functional Magnetic Resonance Imaging) technique in the study of moral intuition, moral emotion, and moral cognition has opened a new page in the study of moral development in recent research (32–36). The neuroscientific approach to morality provides new insights in the study of moral emotion, moral cognition, and moral behavior. The integration of both the affective and cognitive aspects of moral development becomes a meaningful and timely research topic.

In this paper, the characteristics of the moral development of the child will be elaborated with special reference to psychological needs (37), altruism and human relationships (10, 24, 38), and justice reasoning (13–15). While the psychological needs and altruism are related to the affective aspect of moral development, the justice reasoning is related to the cognitive aspect. This is an integrated theoretical model which will combine both the affective and cognitive aspects of moral development together. Prototypic responses to questions related to hypothetical moral dilemmas are presented to substantiate the proposed stage structures.

Perhaps it may be meaningful to delineate the development of the present theory by comparing it with the author’s previous theory (39) published in 1992. The major differences between the present paper and the author’s previous theoretical paper (39) are as follows: (1) in the 1992 paper, the focus is on the moral judgment or justice reasoning and a theory of seven stages was proposed. The present paper, however, is constructed based three parameters: (a) psychological needs, (b) altruism and human relationships, and (c) justice reasoning. The scope is greatly expanded from one parameter (justice reasoning) to three parameters. I have worked out the first three stages of moral development in the present paper and will write another paper on the Stages 4–6 or 7 in the near future. (2) In the present paper, I elaborate the differences among the three stage structures in detail. In particular, the development of each parameter is carefully explained (pp. 41–46). On the other hand, in the 1992 paper I only described the characteristics of each stage but did not delineate the differences between stages in detail. (3) In the 1992 paper, I provided a much more detailed and comprehensive elaboration of the higher stages (Stages 4–7) of justice reasoning. The discussion of the lower stages (Stages 1–3) was less articulated. In the present paper, I have more space to discuss each of the first three stages in a more refined manner, and therefore the whole theoretical structural model is more adequately explained.

## Psychological Needs

According to Maslow’s (38) theory, basic needs are arranged in a hierarchy of pre-potency as follows: physiological, safety, belongingness and love, esteem, and self-actualization. For convenience of discussion, Maslow’s (38) original hierarchy is slightly modified as follows: N1 = physiological and safety needs (i.e., physical survival needs); N2A = love needs; N2B = belongingness needs; N3 = affective and giving needs; N4 = esteem needs; and N5 = self-actualization needs. Two minor rearrangements of Maslow’s original hierarchy are made: (1) the physiological and safety needs are incorporated into one category which may be regarded as the physical survival needs of a person. Broadly speaking, physical survival needs are concerned with biological survival including the maintenance of life and safety. (2) Maslow’s belongingness and love needs are subdivided into three levels: N2A, N2B, and N3. The love needs (N2A) deal with the needs to love and to be loved by the others, in particular by the significant others. The belongingness needs (N2B) refer to the needs to identify and to belong to a primary group such as family, school, religious, or political parties. The affective and giving needs (N3) are concerned with a person’s disposition to love or to show affection to the weak (e.g., a blind person), the good or the elite (e.g., a famous scientist), and the very young (e.g., a child of 6 years old). The esteem needs (N4) refer to the needs for social recognition, social status, and reputation, acceptance by others, and self-esteem. The concept of self-actualization is one of the central themes of Humanistic Psychology. Broadly speaking, self-actualization needs (N5) refer to one’s desire or tendency to actualize or fulfill one’s potential.

It is expected that people at a higher stage of moral judgment should in general show a stronger orientation toward sacrificing their lives for others in an emergency; that is, they should have a weaker tendency to gratify the N1 needs in such situations [(40), p. 263]. On the other hand, the N2A, N2B, and N3 needs are concerned with the love, belongingness, affective, and giving needs which involve altruistic orientations toward significant others, primary group members, and people who are very weak or very good. The gratification of these lower-order needs are so basic and common to all people at different ages that people at different stages of moral judgment should show fairly similar gratification patterns of the N2A, N2B, and N3 needs. As one grows up, one is more oriented toward seeking a higher degree of gratification of higher-order needs (e.g., N4 or N5 needs), and also toward developing higher stages of moral judgment. In other words, a person’s behavior becomes more pro-social and law-abiding as his/her structure of moral judgment develops to the conventional level. Those at the post-conventional level of moral judgment usually abide by the law except in extreme situations where there is a conflict of social law with the self-chosen ethical principles of universal justice (14). Self-actualizers or people with a strong self-actualizing orientation are fair, moral, and democratic people (38), and should be able to reason at the higher stage of moral judgment.

## Altruism and Human Relationships

According to Sharabany and Bar-Tal [(41), p. 50], there is no universally accepted definition of altruism, and the definition varies among theorists of different approaches. With reference to Berkowitz (42) and Krebs (43), Bar-Tal [(44), p. 5] defines altruistic behavior as a voluntary act which must aim to benefit others and which must be carried out without expectation of a reward. A fairly similar definition of altruism is given in Leeds [(45), pp. 230–231], Schwartz and Howard [(46), p. 229], and Wispe [(47), p. 4]. The above definition refers predominantly to self-sacrificial altruistic acts. Some psychologists also regard reciprocally altruistic acts as a type of altruism. Social exchange theorists (48–51) argue that when people interact, they tend to reciprocate with one another in a way so as to maximize rewards and minimize costs. In other words, altruism is either a means for future rewards or a type of social investment. Sociobiologists also argue that reciprocal altruism is naturally selected if the performance of altruistic behavior results in “a return of altruistic behavior toward the original altruist such that the ultimate benefit in units of inclusive fitness is greater than the cost” [(52), p. 94]. On the other hand, it may be argued that reciprocal altruism should not be regarded as a kind of altruism since it is basically selfish. The following criteria for altruism based on Barash (52), Bar-Tal (44), and Leeds (45) are proposed in this study: (1) altruistic behavior must be carried out voluntarily without expectation of a reward. (2) It must aim to benefit the recipient in at least one of the following ways: (a) an increase in the Darwinian fitness, (b) the facilitation of the development of higher stages in cognition, morality, ego, etc., and assistance in attaining new psychological abilities such as intellectual and social skills, (c) an increase in the gratification of basic psychological needs such as physiological, safety, belongingness and love, esteem, and self-actualization needs (38), and (d) assistance in restoring and maintaining emotional stability. (3) Overall speaking, the donor “is doing good” as judged by the recipient.

The study of altruism in psychology is quite extensive (10, 19, 41, 43, 44, 46, 53–56). Psychoanalytic theorists attempt to explain altruistic behavior in terms of attachment. It is argued that man has a strong tendency to form “deep and long-lasting attachments” [(57), p. 129], which greatly intensify altruistic tendencies. Many psychologists [e.g., (58), p. 54; (59), p. 144] regard the Psychoanalytic Theory established by Freud to be the first psychological theory of moral development. On the other hand, behaviorists argue that altruism is a habit or a product of social conformity, which is learned by conditioning process. Social learning theorists (60–63) explain altruistic acts in terms of modeling, positive experience, and observational learning.

Relevant work includes the study of the development of empathy and altruistic motivation (10, 53, 64, 65). However, empirical psychological studies testing part of or the whole of the theory of Human relationships or kin altruism as postulated by sociobiologists are not many. In general, these studies deal with only part of the hierarchy (66, 67).

Based primarily on a synthesis of the work of Barash [(52), p. 316], Carter (68), Hardin [(69), p. 13], the following Hierarchy of Human Relationships is hypothetically constructed in terms of altruism.

### A hierarchy of human relationships

R1: first kin, close relatives.
R2: best friends or intimates.
R3: strangers who are very weak, e.g., a blind person; or very young, e.g., a small child of 6 years old; or who are elite of the society, e.g., a famous scientist who is also a Nobel prize winner.
R4: common strangers.
R5: someone you dislike or enemies.

The main features of the Hierarchy of Human Relationships are as follows: (1) Members in the R1 group are usually genetically related. The terms “coefficient of relationship (*r*)” or “genetic kinship” used by sociobiologists [(69), p. 13; (39), pp. 74–75] are useful for elaboration of this genetic relatedness. Simply speaking, *r* between two persons A and B refers to the proportion of genes in A and B that are identical because of common descent. The *r* between a person (A) and one of his/her parents, son/daughter, or brother/sister is 1/2 and that between A and one of his/her grandparents, uncle, aunt, nephew, niece, and double first cousin is 1/4. In general, the larger (smaller) the *r* between an actor A and another person (B), the larger (smaller) is the probability that A would carry out an altruistic act for B. (2) Generally speaking, a person would value the importance of people in different categories in the following order: R1, R2, R3, R4, R5. In other words, the probability that an actor A would carry out an altruistic act for a person B is the largest if B belongs to the R1 category and decreases consistently to the smallest when B belongs to the R5 category in similar social situations. (3) The above division of the five groups of people is quite arbitrary but the order of the hierarchy: kin – friend – stranger – enemy is invariant of the method of division.

Apart from the sociobiological basis mentioned above, there is also a social basis for the Hierarchy of Human Relationships. Genetically unrelated people who have developed deep affection and profound love between one another may also act altruistically to one another. The tendency for an actor to perform an altruistic act toward a person bearing no genetic relatedness with the actor decreases in the following order of relationships: spouse/lover, best friend, acquaintance, stranger, and enemy. Generally speaking, the interaction between an actor with his/her spouse, lover, or best friend is often pleasant, affective, and frequent. The actor is also familiar with these people and usually bears some essential similarities with them. The interaction between an actor and a stranger or an enemy is, however, less pleasant and less affective.

Ma’s (70) data provided clear empirical support to the above hierarchy of human relationships in two hypothetical dilemma situations. In another study on the relation of altruistic orientation to human relationships and moral judgment in Chinese people, his data supported the following three hypotheses: “(1) the altruistic orientation of an actor at any level of moral judgment is larger to a recipient of closer relationship in any situation; (2) an actor at a higher level of moral judgment would be more willing to sacrifice their life for any recipient than an actor at a lower level of moral judgment; (3) an actor at a higher level of moral judgment would be willing to: (a) give up rescuing a stranger and turn to rescue close relatives or best friends; or (b) help close relatives or best friends by covering up their crime than an actor at a lower level of moral judgment” [(71), p. 377].

In addition, cross-cultural difference in human relationships is an important issue that we need to address. In a cross-cultural study of the hierarchical structure of human relationships, the findings of correlational and factor analysis in both the London and Hong Kong Studies supported the proposed hierarchical structure of human relationships from R1 to R5 (72).

### A mathematical formula of human relationships

Let the tendency of carrying out an altruistic act by an actor toward a person having R*_i_* relationships with the actor be R*_i_*, then:

(1) $$R_{i}>R_{j}\;\mathrm{for}\;1≦\;i<j\;≦5\;\mathrm{and}\;i\neq j.$$

In other words, the altruistic orientation decreases consistently from R_1_ (kin and close relatives) to R_5_ (enemy or someone you dislike). The relationship between R*_i_* and R*_j_* can also be studied by considering the correlations between R*_i_* and R*_j_* for a sample of participants.

Let R(*i j*) = (R*_i_* R*_j_*) = R(*j i*) = (R*_j_* R*_i_*) = Correlations between R*_i_* and R*_j_*.

(2a) $$\begin{array}{ll} & R\left(i\;j\right)<R\left(i\;k\right)\;\mathrm{for}\;5≧\;j>k>i\;\mathrm{where}\;3≧\;i≧1 \end{array}$$

(2b) $$\begin{array}{ll} & R\left(i\;k\right)>R\left(j\;k\right)\;\mathrm{for}\;1≦\;i<j<k\;\mathrm{where}\;5≧\;k\;≧3 \end{array}$$

From Eq. (2a), we have the following:

$$\begin{array}{llll} & R\left(15\right)<R\left(14\right)<R\left(13\right)<R\left(12\right)\; & & \;\\ & R\left(25\right)<R\left(24\right)<R\left(23\right)\; & & \;\\ & R\left(35\right)<R\left(34\right)\; & & \; \end{array}$$

Similarly, from Eq. (2b), we have the following:

$$\begin{array}{llll} & R\left(15\right)<R\left(25\right)<R\left(35\right)<R\left(45\right)\; & & \;\\ & R\left(14\right)<R\left(24\right)<R\left(34\right)\; & & \;\\ & R\left(13\right)<R\left(23\right)\; & & \; \end{array}$$

That is, R(*i j*) increases toward the diagonal along a column or a row in the correlation matrix of R(*i j*); or R*_ij_* decreases away from the diagonal along a column or a row. The R(*i j*)’s can only be studied at group level.

## Justice Reasoning: The Cognitive Developmental Approach to Morality

The structure of judgment is another important aspect of moral development, and the study of moral judgment is probably the most popular research topic in the field of moral development. A number of theories have been proposed (13–15, 73). The study of social cognition is also relevant in this case (74–76).

Although the major influence on the Cognitive Developmental Approach has been the early work of Piaget (13), this approach has roots in the work of the theorists Durkheim (77), Paul Fauconnet and Pierre Bovet (See (13), Chapter 4). Also, Dewey (78, 79) with his theory of conduct and experience, Mead (80) with his theory of role-taking, Loevinger (24, 81) with her theory of ego development and Rawls (82) with his theory of justice have influenced the theoretical development by Kohlberg (14, 15) and Rest (20, 83).

### Piaget’s two major stages: Heteronomy and autonomy

In his studies, Piaget identified two major stages of moral judgment. The earlier stage which occurs before the age of seven or eight is called Heteronomy, moral realism, or a morality of constraints. The later stage is called Autonomy or a morality of co-operation. Piaget (13) asserts that all children develop their moral judgment from the Heteronomy stage to the Autonomy stage. In addition, there is a premoral stage before the Heteronomy stage. However, the thought processes underlying these two major stages are partially overlapping. In addition, the Autonomy stage “gradually succeeds in dominating” the Heteronomy stage. In other words these two moral attitudes “may co-exist at the same age and even in the same child, but broadly speaking, they do not synchronize” (p. 129).

In addition, Piaget [(13), pp. 407–411] postulates a parallelism between the child’s moral judgment and his intellectual development. The young child’s morality of constraint is accounted for by two major factors. The first is the child’s egocentrism or his logical incapability to distinguish “what belongs to things and other people from what is the result of his own particular intellectual and affective perspective” (p. 407). The second is the child’s unilateral respect for adults “From the intellectual point of view, this respect gives rise to an ‘annunciatory’ conception of truth” (p. 408). Moreover, it causes the young child to treat moral rules as external, ready-made, and unchangeable. As the intelligence of the child develops from pre-operational thinking to operational thinking, he begins to realize the spirit of co-operation, the principles of reciprocity and equality, the third-person perspective, and concern for the welfare of others. He then transits from the Heteronomy stage to the Autonomy stage.

### Kohlberg’s theory of moral development

The conception of this parameter was based mainly on Kohlberg’s (14, 15) theory of moral judgment development which is concerned with structures of moral thinking about interpersonal conflict situations. Kohlberg postulates a sequence of six distinct moral stages, which is invariant for all persons. In particular, Kohlberg (14, 15) applies quite extensively Kant’s moral philosophy and Rawl’s (82) theory of justice in the elaboration of his Stage 6. The principles of justice defining Stage 6 are said to be content-free, self-chosen, and non-contingent upon any prior agreement or contract. The only assumption is that it applies to all human beings. In other words, all persons are treated as morally equal, and commutative justice is regarded as reciprocity, contract, and trust. Kohlberg argues that the principles of justice in his Stage 6 satisfy the formalist requirement that rational moral judgment must be reversible, consistent and universalizable. Kohlberg (84) also claims that “no principle other than justice has been shown to meet the formal conception of a universal prescriptive principle” (p. 221). He finally argues that no other concept of morality is stronger and more positive than this one.

In a critical review of 45 empirical studies of moral judgment development carried out in 27 countries, Snarey (85) concluded that Kohlberg’s (14, 15) Stage 1 to Stage 3/4 or 4 could be regarded as universal. In addition, it was also concluded that “although the presence of Stage 4/5 or 5 was extremely rare in all populations, it was evident to some degree in approximately two thirds of the subcultures sampled that included subjects in the 18–60 age range” [(85), p. 226]. Earlier reviews of cross-cultural studies of Kohlberg’s stages by Bergling (86) and Edwards (87) also remarked that the higher stages (5 or 6) were not universal.

Krebs and Denton (88) argued that the cognitive developmental approach to morality by Lawrence Kohlberg is inadequate and has come to the end of an era. They proposed a pragmatic theory of morality to account for everyday moral behavior. Gibbs (89) did not agree to Krebs and Denton’s argument. He argued that “evidence suggest that Krebs and Denton may have underestimated relations between moral judgment stages and social behavior, including sudden behavior in emergency situations” (p. 666).

## Hypothetical Dilemmas

In order to illustrate the feature of the three stages of moral development, responses given by subjects to the following hypothetical dilemmas constructed by the author (90–92) are reported. For each of the hypothetical dilemmas, subjects first make a response to each of the X’s on a 7-point scale: definitely yes, strongly yes, moderately yes, can’t decide, moderately no, strongly no, and definitely no. Afterward, a number of open-ended questions regarding the reasons for their decision are asked.

### A lost bag

Suppose 1 day, when you are walking by yourself along a road, you discover a bag accidentally. You open it and find that the bag contains a lot of money, almost $100,000, and some documents showing that the money belongs to a big company. It so happens that for a particular reason X, you need a great deal of money immediately and *there is no other way* for you to obtain such a large amount of money except by keeping the money in the bag. Would you do so if the reason X is …?

X1 = you have been accepted by a world-famous university abroad for a 2-year course which you earnestly desire to attend. However, there are no grants or scholarships available and the cost each year is about $50,000.
X2 = you are near death from a rare disease, which the doctors think may be cured only in a particular hospital in another country. The total expense will be about $100,000.
X3 = suppose it is a sister or brother who has the rare disease in the above case.
X4 = you recently started your own business and in the past 2 years you have borrowed $100,000 from a bank. The bank manager tells you that you have to return all the money in two weeks’ time because of unpaid interest, otherwise he will have to prosecute you.
X5 = suppose in the case X4, it is your best friend who has borrowed $100,000 from the bank for his or her business. Sometimes ago, you promised your friend that you would help him/her by all means when in need. Now your friend asks you to lend him/her $100,000.
X6 = you want to buy a luxurious car for yourself.

### The sinking boat

You and X are in a boat which is sinking, but only you *or* X can be rescued. Would you sacrifice yourself so that X could be rescued if X is …?

X1 = a young stranger, 20 years old; X2 = an old stranger, 70 years old; X3 = a famous scientist who is also a Nobel prize winner; X4 = your brother or sister; X5 = your best friend; X6 = a postman; X7 = someone you don’t like or an enemy; X8 = a child, 6 years old; X9 = your husband/wife.

### A doctor’s dilemma

Susan is a young medical doctor. She worked in a private hospital which had many rich patients and she earned a good salary. She is engaged to marry Peter who is also a doctor in the same hospital. However, Susan had not felt very happy with her job over the last few years and decided to go to work as a voluntary doctor in a poor and underdeveloped country for at least 5 years. Her parents, Peter and her friends all objected strongly to her decision. Nevertheless, she managed to overcome all the difficulties and is now working in a remote village where she is the only doctor. Susan feels very happy about her present job and is highly respected and loved by the villagers. After 2 years’ work in the village, Susan is faced with a difficult problem X which has to be resolved either by staying or leaving and returning to her own country. *Suppose you were Susan*, would you *give up* the present job in the village if the problem X is …?

X1 = her fiancé Peter wrote to her and said that he would not wait any longer. If she is not going to return within a few months, he will not marry her.
X2 = her mother has suffered a stroke and is paralyzed. She wants Susan to come back and look after her.
X3 = because of the economic recession, the voluntary organization she is affiliated to has to stop the medical supplies to her clinic this year. The only way to maintain the clinic is to borrow a large amount of money from her friends.
X4 = because there are troubles in the country, and terrorists are still in the bush and attack remote villages. Susan’s village and clinic have been attacked twice already. Fortunately, she was not injured. Now her village is under constant threat.
X5 = because of her failure to save the life of the son of the headman of the village, the headman is very angry and is going to force her to leave the country. The only way is to organize the villagers to stand on her side, which is obviously difficult to do.
X6 = many countries, including Susan’s original one, are suffering from plague. Susan receives a letter from her own government, asking her to report for duty immediately. However, the plague also starts to spread in the village she is working in.

## The Moral Development of the Child

The stages of moral development of the child with special reference to psychological needs, altruism and human relationships, and justice reasoning are constructed. The focus is on the moral development of kindergarten and elementary school (Grade 1–6) children.

### Stage 1: Physical survival, selfishness, and obedience

#### Psychological beeds: physical survival

The major emphasis of this stage is on physical survival. People at this stage would argue that it is right to be selfish for the sake of physical survival.

##### Survival and safety orientation

People at this stage place emphasis entirely on their physical survival and safety needs (38). What is right is to do things that would favor their gratification of survival and safety needs, very often at the expense of others. In other words, they tend to place more emphasis on materialistic and lower needs rather than spiritual and higher needs. In short, they tend to be very selfish and egocentric. The struggle for food, water, sexual satisfaction, and materialistic awards is their major concern.

People at this stage would act by all means to get what they want or to satisfy their physiological and safety needs. On the other hand, they quite fear being physically hurt or physically punished. They also fear death, nightmare, being caught, or being jailed. A 7-year-old boy responded to “The Sinking Boat Dilemma” as follows (Q = Question and A = Answer):

Q: Would you try to rescue others in this situation?

A: Yes.

Q: Why?

A: If I don’t rescue him, I am afraid of dreaming (during my sleep).

Q: What sort of dream?

A: Dreaming of ghosts (nightmare).

The subject’s major concern in this case is his fear. He is willing to help because he is afraid of the negative consequence of not offering the help. Another 7-year-old girl responded to “A Lost Bag” dilemma as follows:

Q: Would you take away the money in the lost bag to cure your disease?

A: I would take away the money in the bag to cure my disease because I fear death.

Q: How about to pay back the debt to the bank?

A: I would also take the money to pay back my debt because I fear being jailed and I fear that I couldn’t see my father, mother, sister, cousins, and auntie.

The fear of death, being jailed, and being separated from one’s significant others is the main motivation for taking away the money from the lost bag. In other words, the subject would be willing to take away another’s money in order to reduce or to remove his or her fear.

A 14-year-old boy responded to “A Lost Bag” dilemma as follows:

Q: Would the chance of being caught by the police affect your decision to take away the money in the bag?

A: The chance of being caught by the police would affect my decision to take away the money in the bag because I might be caught and jailed, and anyway I dare not to use the money.

The subject is very realistic and he would not take away the money from the lost bag to solve his problems if the chance of being caught by the police is high. Anyway, it is not safe to do so and he dares not to do that.

An 8-year-old boy gave a very interesting response in “The Lost Bag” dilemma as follows:

Q: Generally speaking, is it your responsibility to take the lost bag to the police?

A: Yes. If I don’t take the money bag to the police, I would feel uneasy and have nightmare. “Hey, kid, why do you take away the money bag?” That person would say to me like that in my dream. Or he would catch me and put me into jail. The money doesn’t belong to me. The subject felt that it is not safe and it is disturbing to take away the money bag.

##### Pleasure principle

People at this stage tend to emphasis on seeking personal pleasure, fun, happiness, and comfort. For matter of physical survival and personal safety, they follow the principle of pleasure in their behavior, especially in their interaction with authorities. A 6-year-old girl gave the following response in “A Doctor’s Dilemma” as follows:

Q: (The village is under the attack by terrorists.) Why would you definitely give up the present job in the village if you might be attacked by terrorists?

A: Terrorists, I am afraid of. I will go home once every year. If being attacked and I don’t go home, my parents will be very worried about me (about my safety).

The subject is clearly concerned about her safety and home is a place where she found comfort and safety.

#### Altruism and human relationships: selfishness and attachment to parents

People at this stage are in general selfish. They have deep and profound attachment to their parents and usually act altruistically in order to please their parents or authorities.

##### Empathy to significant others

People at this stage can imagine themselves in another’s situation and therefore are able to exhibit empathy in response to other’s distressful feeling. The empathetic response is deeper and more profound to significant others such as parents. A 6-year-old boy responded to “A Lost Bag” dilemma as follows:

Q: Why would you take away the money in the bag to cure your brother’s disease?

A: Because he is very miserable, need to cure him.

Q: Suppose the money bag belongs to a person who is in similar situation, would you return the bag to him?

A: Yes. He is very miserable and needs the money.

The subject exhibited a deep empathy to his brother as well as to the person in similar situation. It is a simple and straightforward response to the miserable suffering of others. The same subject responded to “A Doctor’s Dilemma” as follows:

Q: Why would you definitely give up your present job in the village to take care of your mother?

A: Mummy is very pitiful, and very painful.

Q: (The village is under the attack by terrorists.) Why would you definitely give up the present job in the village if you might be attacked by terrorists?

A: Because of fear of death, a horrified death.

The response to the first question exhibits a deep empathy to his mother. On the other hand, the answer to the second question here is clearly concerned with survival needs.

##### Selfish orientation

People at this stage are selfish. They tend to seek pleasure and avoid pain, very often at the expenses of others. It is their intention to get as much as possible from others but they tend to refuse to benefit others or society. An 8-year-old boy responded to “The Sinking Boat” dilemma as follows:

Q: Suppose X is a pet you like (e.g., a cat, a dog, or a rabbit), would you sacrifice yourself for X?

A: I would rescue the rabbit.

Q: Why?

A: It’s funny, I can keep it.

Q: Suppose we compare a pet with an enemy, would you sacrifice yourself for an enemy or a pet? Why?

A: I would save the rabbit because it’s funny. I wouldn’t save the enemy because he is a bad guy.

The subject is only able to argue from his egocentric viewpoint: he likes to keep a pet and it is funny to do so. He would not save the life of an enemy because he is a bad guy. The subject’s viewpoint is self-centered, selfish, and limited in scope.

##### Affective bonding: attachment to parents

The symbiotic relation is a dominant characteristic of attachment in early childhood [(24), pp. 15–16]. It can be summed up as “If mother cries, I cry. If father is unhappy, I am unhappy too.” The non-separate relation between parents and the individual makes the person act almost entirely according to the wishes of the parents. While the symbiotic attachment is not a major feature of this stage, the attachment between the son/daughter and the parents is still deep and profound. The sons/daughters will act altruistically toward their parents just as they would treat themselves. A 9-year-old girl responded to “A Doctor’s Dilemma” as follows:

Q: Would you give up your present job in the village to take care of your mother?

A: (Strongly yes.) Because Mummy is sick, I need to cure her. If she dies, then I and my fiancé would be left alone, very pitiful, very lonely.

The subject shows a profound attachment to her mother. She would be willing to sacrifice her job in order to take care of her mother.

A 8-year-old girl responded to “A Lost Bag” dilemma as follows:

Q: Would the chance of being caught by the police affect your decision to take away the money in the bag?

A: Yes. If I was caught by the police, my mother would be left alone at home, this is not good.

The subject is very much concerned about the attachment between her and her mother.

##### Authority-induced altruism

People at this stage would act altruistically only to gain approval or to avoid punishment by authorities [(14), p. 17; (41), p. 67]. They would take care of a small group of significant others such as their parents who exert considerable influences on their daily life. A 7-year-old boy responded to “A Lost Bag” dilemma as follows:

Q: Generally speaking, is it your responsibility to take the lost bag to the police?

A: Yes, (my) teachers teach me to help others, we have to listen to them carefully. I don’t quite understand why (I should help others).

The subject complies with the teaching of the authority, in this case his teachers, that one should help others. Another 9-year-old girl responded to “The Sinking Boat” dilemma as follows:

Q: Generally speaking, is it your responsibility to help or rescue a person in danger?

A: Yes. I don’t know why it is my responsibility. (But) Mummy said that if someone has difficulty, we should help.

The subject does not understand what it means by responsibility but she would follow her mother’s teaching to help those in need.

#### Justice reasoning: obedience to authority and heteronomous morality

People at this stage obey blindly what the authority commands in order to avoid punishment.

##### Obedience to authority

One main reason for a person to obey what the authorities command is to avoid physical punishment. Other reasons identified by Piaget (13) and Lickona (3) include the following: (a) Unilateral respect to parents: it refers to the one-sided respect paid by a child to his or her parents or authorities in the process of conforming his or her behavior to the adults’ constraints. (b) Immanent Justice: it refers to the belief in “the existence of automatic punishments which emanate from things themselves” [(13), p. 250]. The basic assumption of the principle of immanent justice is that natural forces are always in the hands of adults and ensure that the disobedient will be punished. (c) The child takes the assumption that moral rules are external and rooted in adults and authorities. It follows naturally that those who are punished by adults must have done something wrong. (d) The child believes in expiatory or arbitrary punishment. He thinks that “the only way of putting things right is to bring the individual (wrong doer) back to his duty by means of a sufficiently powerful method of coercion and to bring home his guilt to him by means of painful punishment” [(13), p. 203]. The form of punishment can be arbitrarily determined by adults. (e) The child believes in retributive justice which means that each person should be awarded according to the arbitrary and unequal distribution of rewards by adults. (f) The child believes that one should not take one’s revenge because there is a more legitimate way – to report the aggression to adults and they would punish the aggressor fairly.

A 7-year-old girl responded to “A Lost Bag” dilemma as follows:

Q: Generally speaking, is it your responsibility to take the lost bag to the police?

A: Yes. Because greed is not good, my teacher said that.

Q: Would the chance of being caught by the police affect your decision to take away the money in the bag?

A: Definitely no. Because greed is not good, I would also be caught by the police.

Q: Would you take away the money from the bag to cure your disease?

A: Definitely no. Because greed would be condemned by mother and other family members, and I would be caught and sent to the police station.

The subject obeyed the commands of authorities (teacher and mother) and thought that one should not be greedy. A 9-year-old girl responded to the same dilemma as follows:

Q: Generally speaking, is it your responsibility to take the lost bag to the police?

A: Yes. Teachers teach (us), (we) should take it to the police.

What the teacher teaches will be followed closely, a common expression of obedience to authority in children.

##### Egocentric viewpoint

People at this stage often find difficulty in understanding differences in points of view between themselves and others. In other words, they are not aware of other’s reasoning from a third-person perspective. They also confuse the authority’s perspective with their own. An 8-year-old boy responded to “A Lost Bag” dilemma as follows:

Q: Do you love your sister?

A: Yes.

Q: Why is that you wouldn’t take away the money in the bag to cure your sister’s disease?

A: Mother would give (the money to cure the sister’s disease).

Q: How about if mother has no money?

A: Father would give.

Q: How about if neither father nor mother has money?

A: Father definitely has the money.

The subject is very egocentric in his argument. He thinks that his parents would definitely have the money to solve the dilemma and ignores the interviewer’s questions.

##### Rigid social norm and unchangeable rule

People at this stage regard rules, social norm, traditional values, and common practices as rigid and unchangeable guidelines for their behaviors. What is right is to comply as closely as possible with social norms and practices. For example, filial piety is regarded as the most important value in some Oriental societies, and people at this stage would obey their parents and follow their parents’ wishes above all else. It would be totally unacceptable to do things against the wishes of their parents. For young children, rules are fixed and unchangeable. They would not change a rule because of the intention of the actor or the unexpected situational variables. “A rule is therefore not in any way something elaborated, or even judged and interpreted by the mind; it is given as such, ready-made and external to the mind” [(13), p. 106]. A 7-year-old girl responded to “A Lost Bag” dilemma as follows:

Q: Suppose the money in the bag belongs to a person in a situation similar to you, would you return the money to him?

A: Yes. Because if I have used other person’s thing, I must return. For example, if I have borrowed other’s ruler, I have to return after use. If you don’t do so, other people would regard you as a thief.

The rule is that you must return the thing you borrow because it is not your property, otherwise you are a thief. This rule must be abided by and cannot be changed. Therefore, we need to return the money bag to the owner because it is not ours. Another 8-year-old girl responded to the same dilemma as follows:

Q: Why wouldn’t you take away the money in the bag to cure your disease?

A: I would feel uncomfortable.

Q: Suppose the money bag belongs to a person who is in similar situation (i.e., the person is sick), would you return the bag to him?

A: Yes, the money belongs to him.

Q: Why wouldn’t you take away the money in the bag to cure your brother’s or sister’s disease?

A: Greed is not good. Even though you use (the money), you may not cure the disease.

Q: Suppose the money bag belongs to a person who is in a similar situation (i.e., his/her brother or sister is sick), would you return the bag to him?

A: Yes, the money belongs to him.

Q: Why wouldn’t you use the money in the bag to help your friend to pay back the bank?

A: The money belongs to other person. The money is not mine, so I could not give it to other person (my friend).

The norm is that if the thing is not yours, you must not keep it; and you should return it to the owner. This subject sticks consistently to this norm in his responses to several questions.

### Stage 2: Love needs, reciprocal altruism, and instrumental purposes

#### Psychological needs: needs for love and companionship

The major focus of this stage is on needs for love, affection, and caring as well as needs for companionship, partnership, and friendship.

##### Love needs

People at this stage focus on one’s love needs and self-happiness They are also quite self-protective. A 7-year-old boy responded to “A Doctor’s Dilemma” as follows:

Q: Would you give up the present job in the village if your fiancé asks you to go back? Why?

A: I would go back and explain to my fiancé, cook nutritious food for him to eat, and stay there with him. I would not return to the village because staying with him is very happy.

The subject is not interested in helping the patients in the village. He is more concerned about love needs. In other words, he placed more emphasis on the doctor’s love relationships with her fiancé than her self-actualizing act in the village. Another 8-year-old boy responded to “A Lost Bag” as follows:

Q: Why would you take away the money in the bag to cure your disease?

A: If I have terminal disease, I would take the money to cure my disease so that my dad and mum would not worry about me.

Q: Why would you take away the money in the bag to cure your brother’s disease?

A: I don’t want to lose one brother. Dad and mum would worry that my brother is going to die.

The subject is concerned about the deep attachment and profound love between himself and his parents, and also between his brother and his parents. He would use the money in the lost bag to cure his disease as well as his brother’s disease so that his parents would not feel unhappy and worried.

##### Needs for companionship

The need for companionship, partnership, and friendship is the major feature of this stage. People at this stage emphasize caring, affective, and love relationship more than other things such as wealth and reputation. They treasure the affective and intimate relation with their spouse, lover, partner, and good friend.

A female undergraduate responded to “A Doctor’s Dilemma” as follows:

Q: Would you give up practicing medicine in the village if your fiancé threatens not to marry you in case you do not return to reunion with him right away? (The subject’s answer is moderately yes). Why?

A: From a female perspective, I emphasize marriage more than career. However successful you are in career, you need to get married. My fiancé has already waited for me for quite some time, which has at least proved that he really cares me. If you give up this opportunity, you may not get someone as good as him afterward.

The subject is willing to give up her self-actualizing act in order to maintain the affective relation with her fiancé. The desire for affective relation and the emphasis on marriage is a common value held by many people.

#### Altruism and human relationships: reciprocal altruism

People at this stage regard reciprocal altruistic acts as the rights acts. They exhibit more profound empathy and altruism toward lover, intimates, and closely related people.

##### Empathy toward lovers, intimates, and closely related people

People at this stage are able to think and feel from the perspective or role of the other. They can put themselves in others’ shoes and react empathetically to others’ feeling of unhappiness, sadness, pain, distress, discomfort, and loneliness; and therefore have the motivation to help these people. But they exhibit more empathic distress toward lovers, intimates, and closely related people such as siblings and relatives. A 14-year-old Grade 8 girl responded to “A Lost Bag” dilemma as follows.

Q. Would you take away the money in the bag to cure your brother or sister’s disease? (The subject’s answer is moderately yes). Why?

A: Because my brother or sister was facing death, very painful and miserable, and I could not bear to see them suffer. In addition, the family needs their financial support, therefore I would take the money to rescue them.

The subject is willing to help her sibling because of affection and for instrumental purposes. She is empathetic about her sibling’s suffering and is also concerned about the fact that her family requires her sibling’s financial support. A male undergraduate subject responded to “A Lost Bag” as follows:

Q: Would you take away the money in the bag to cure your brother or sister’s disease? (The subject’s answer is moderately yes). Why?

A: Because he (or she) is my brother or sister. Although it seems that it is not quite morally right but I can’t tolerate to see my brother or sister to die because of lacking money to cure the disease.

These two examples are relevant to Hoffman’s (10) “Friendship Bias” (p. 207). That is, people express more empathic distress toward friends, intimates, and closely related people.

##### Reciprocal altruism

People at this stage understand that other people also have similar needs and interests as themselves and therefore they regard reciprocally altruistic behavior as a desirable act. The idea of reciprocal altruism is clearly expressed in the following two descriptions: (a) “You scratch my back and I’ll scratch yours,” and (b) “You help me today and I will help you tomorrow in return.”

A 6-year-old girl responded to “The Sinking Boat” dilemma as follows:

Q: Generally speaking, is it your responsibility to help or rescue a person in danger?

A: Yes, because if you don’t save him, he would die. Saving his life is helping him, he would thank my parents later on.

Q: How to thank (your parents)?

A: Giving them gifts.

The subject thinks that if she sacrifices her life to rescues a person, the recipient will reciprocally give gifts to her parents. This is a fairly complicated case of reciprocal altruism because the benefit is not received directly by the altruist herself. The benefit is given to the altruist’s parents, the significant others of the altruist.

An 8-year-old boy responded to “A Lost Bag” dilemma as follows:

Q: Why couldn’t you decide whether you would take away the money in the bag to cure your disease?

A: I don’t know.

Q: Suppose the money bag belongs to a person who is in a similar situation, would you return the bag to him?

A: Yes. When he recovers, he can help me to borrow money to cure my disease.

Q: Why would you take away the money in the bag to cure your brother’s or sister’s disease?

A: I cure (help) him, and he would recover. When I am sick, he can then help me in return.

Q: Suppose the money bag belongs to a person who is in a similar situation, would you return the bag to him?

A: Yes, he would recover. I would then ask other people to lend me money (to cure my brother or sister’s disease).

The subject thinks that it is important to help others because they would help you back in the future. This is another clear-cut case of reciprocal altruism.

##### Friendships and peer relationships

The affection and caring at this stage extends to siblings, best friends, intimates, and lovers. The relationship is a horizontal and reciprocal one. People at this stage not only build up an affective relation with their parents but also with their siblings and selected groups of people of similar age and interests. A 6-year-old girl responded to “A Lost Bag” dilemma as follows:

Q: Why would you definitely take away the money in the bag to help your friend to pay back his debt to the bank?

A: Friends should help each other. Otherwise, they are not good friends.

Q: But you may be jailed (because of taking away the money in the bag)?

A: I go to jail and not him (her friend), I would feel comfortable. (Doing a favor) For him, it is OK.

The main argument is that good friends should help each other, even though one has to bear the risk of being put to jail. It is better to suffer oneself than for a friend to suffer. The altruism for friends is quite profound and salient in this case.

A 7-year-old boy responded to “The Sinking Boat” dilemma as follows:

Q: Generally speaking, is it your responsibility to help or rescue a person in danger?

A: Yes. Helping others is very good. If you help others, they would help you in return. I would rescue friends and wouldn’t rescue those I don’t know.

The subject makes it clear that he would perform altruistic acts toward his friends but not to strangers.

#### Justice reasoning: instrumental purposes and opportunistic hedonism

People at this stage tend to act in their own self-interests. However, they also have “a clear sense of fairness as quantitative equality in exchange and distribution between individuals” [(14), p. 148]. According to Kohlberg [(14), p. 148], the idea of equal exchange can be expressed by the following statement, “you shouldn’t hurt or interfere with me, and I shouldn’t hurt or interfere with you.” In addition, they tend to use all means, whether it is legitimate or not, in maintaining their survival and getting what they want.

##### Instrumental purposes

Acts are usually regarded as instrumental means to serve one’s needs and interests. For instance, people at this stage tend to help others who are in desperate situations because they expect others to do the same for them some day. On the other hand, if the situation does not clearly indicate that such help would bring them more benefits than cost to the actor in the long run, then the actor would stick to the rule “mind your own business” or “let things drift if they do not affect one personally,” and would not act to help the victims. The contents of the exchange or the deal are often concrete or materialistic things such as money or food, or things which are perceived as good to serve one’s own needs or interests such as praise from authorities. The perspective of judgment is individualistic; self-interests precede group or others’ interests. It should be noted that things that are too general or abstract such as basic rights of human beings are seldom considered or valued in the exchange or deal.

A 7-year-old girl responded to “The Sinking Boat” dilemma as follows:

Q: When you decide to rescue for X, would you consider X’s occupation or educational level?

A: I would rescue the teacher first. The teacher would teach us how to read books, if (I) don’t rescue him, no one would teach us to read.

The subject’s decision to rescue a person is clearly based on instrumental purpose. She would rescue someone who would benefit her in one way or another. And in this case, the subject thinks that the teacher is important and should be rescued because she needs the teacher to teach her to read.

##### Opportunistic hedonism

Since people at this stage are holding a concrete individualistic perspective, the positive claims or welfare of others are in general not their concern or responsibility unless such claims and welfare are part of the exchange or deal. In other words, “one has a right to ignore the positive claims or welfare of others as long as one does not directly violate their freedom or injure them” [(14), p. 215].

In addition, people at this stage also believe that “life is a zero-sum game; what one person gains, someone else has to lose” [(24), p. 17] It is of course better to gain for oneself and to let others lose. To put it in an extreme form, it means that it is better for others to die and for me to live, if necessary. In other words, they are Machiavellian in maintaining their survival and getting what they want. That is, in order to survive or to get what they want, they would consider using any means, whether the means is legitimate or not. In addition, “work is perceived as onerous. The good life is the easy life with lots of money and nice things” [(24), p. 17]. The idea is that one should try to get a lot just by making little or no effort. Generally speaking, people at this stage claim as much rights as they can but tend to bear as little responsibilities as possible. In other words, they act or survive by the principle of opportunistic hedonism.

##### Norm of equal exchange

The compliance to social norms, propriety and common practices is based on instrumental purpose and equal exchange. For example, keeping promise is a propriety or a norm in a society. But one would think that it is right not to keep a promise to a person who has not kept a promise to him or her in the past. If anyone does not comply with the norm, people at this stage would think that it is right to revenge or retaliate. In other words, “an eye for an eye” or “if you hurt me once today, I will hurt you twice in return tomorrow” is acceptable. A 17-year-old boy responded to “A Lost Bag” dilemma as follows:

Q: Would you take away the money in the bag to help your friend to pay back his debt to the bank? Why?

A: Strongly yes. Because I have promised my friend to help him when he is in need. And now I have picked up the money bag by chance, so I would use the money to help him. More important, we are friends.

Q: Keeping promise and abiding by the law, which one is more important? Why?

A: If today I keep my promise and help my friend. Perhaps 1 day, when I need help, he would help me. …. And if (I) abide by the law today and don’t help my friend, then when I need help in future, my friend would also not help me. So (if I) abide by the law today, it is not beneficial to me. But I realize that abiding by the law is right.

The subject realizes that it is right to abide by the law but he chooses to keep the norm of promise instead of abiding by the law because of the equal exchange and reciprocity with his friend. In addition, he regards keeping promise to friends is an important rule to follow but apparently appears to fail to understand the importance and meaning of abiding by the law.

### Stage 3: Belongingness needs, primary group altruism, and mutual interpersonal expectations

#### Psychological needs: family belongingness and group identity

The characteristics of this stage include a desire to build up one’s own family. The needs for belongingness are the basis for making their moral decision.

##### Belongingness needs

The decision for an action is based on one’s needs for belongingness and love. In other words, one has a strong need to attach to a primary group. Primary group refers to family, gang, group of friends or intimates, club, school, party, organization, company, etc. Generally speaking, members of a primary group share common interests, philosophy, ideology, and in some cases property. A female undergraduate responded to “A Doctor’s Dilemma” as follows:

Q: What do you think is the most important thing a son should be concerned about in his relationship to his father?

A: The son should be filial to his parents. The son should not disappoint his parents, and the parents should love and care about their kids.

Q: Why is that the most important thing?

A: To maintain the family harmony depends on mutual respect. Otherwise if we don’t have affection, we won’t be happy and we won’t have sense of belonging.

The subject’s response demonstrates a typical example of primary group affection and family cohesion. The sense of belonging to the family is the major concern of the subject. Another female undergraduate responded to “The Sinking Boat” dilemma as follows:

Q: When you are making a decision to sacrifice yourself for X (others), would you consider whether X would promise to try his or her best to take care of your family after your death?

A: Yes. If I am facing death, and there is no one taking care of my family, I would be very worried. If the recipient can take care of (my family) or is even more caring than myself, I would be easier.

The subject shows a deep and profound affective orientation toward her family.

##### Group identity

People at this stage emphasize group conformity and group loyalty. They regard the group identity as an important factor in making their moral decision when they face dilemmas. An undergraduate subject responded to “The Sinking Boat” dilemma as follows:

Q: When you try to rescue a person, does the race of the person matter?

A: I would choose to rescue my country’s people.

Q: Why?

A: Closer. I think, our blood relation is closer, (and) better.

People at this stage tend to rescue their country’s people first because of the group identity and close blood relation. In addition, the maintenance and protection of the esteem, dignity, reputation, and social status of the primary group is emphasized at this stage. The individual’s esteem need is tied with the esteem of the primary group.

#### Altruism and human relationships: primary group altruism

People at this stage are more altruistic to members of their primary group than to out-group members. The Hierarchy of Human Relationships (Kin/close relatives – Best friends – Strangers) and family affection are emphasized at this stage.

##### Empathy toward primary group members

People at this stage show deeper empathy for members of the primary group than for external members. In other words, they feel much more distressed about the suffering of those closely related with them than those who are less related with them. This is exactly what Martin Hoffman (10) called “Familiarity Bias” or “In-group Bias” (pp. 206–207).

An undergraduate responded to “The Sinking Boat” dilemma:

Q: Close relatives, good friends, and strangers; is your willingness to sacrifice for them the same? (The subject’s previous responses indicated that the willingness to sacrifice for close relatives and best friend is much higher than that for the stranger). Why?

A: I think this is normal human affection. You would be more willing to sacrifice for those you have affection. It would be much more difficult to bear that fact that you live and the other dies if the other person you are acquainted with.

Q: Do you think this behavior is a selfish one?

A: I don’t think this is selfish, this is normal human affection.

Q: Why?

A: For someone you are acquainted with, you would care more. That is, you would care his death more. This is a very natural thing, and it is therefore a kind of normal human affection.

The emphasis is on the much deeper empathy that you would have for those you are acquainted with in comparison to strangers. “Those you are acquainted with” include close relatives like parents, closely related people, and best friends; that is, members of the primary group. In other words, it would be much more difficult to bear the suffering of the primary group members than that of the other people. A similar and interesting response was given by a 17-year-old boy in “The Sinking Boat” dilemma:

Q: Close relatives, friends, and strangers: is your degree of sacrifice for them the same? Why?

A: No. Rescue relatives first. Because seeing relatives, friends, and strangers die, you would feel sorrowful and miserable in your heart. But seeing relatives die is the most sorrowful; friends, less so; strangers, just a little bit uncomfortable.

Q: Is it a natural thing?

A: Natural. Everybody’s decision is the same. It is a natural law.

The subject exhibits a variable degree of empathy toward others. The intensity of empathic distress decreases consistently in the following order: close relatives, friends, and strangers.

##### Primary group altruism

People at this stage are willing to perform altruistic acts toward in-group members at great personal sacrifices. However, they are much less willing to do so for out-group members. They consider the gratification of the basic needs of the primary group when they face a dilemma situation. When both they themselves and the primary group are in the state of deficiency of basic needs, they tend to regard the interests of the primary group as being as important as theirs, or at least as the next most important. An example of affection and empathy toward sibling and parents (i.e., members of a family) is given by a 15-year-old girl in “A Lost Bag” dilemma.

Q: Would you take away the money in the bag to cure your disease? Why?

A: Definitely no. Even though I take away the money in the bag to cure my disease, it is only possible to cure it. Human being will die any way. Since I have terminal disease, why bother to cure it?

Q: Why would you definitely take away the money in the bag to cure your brother or sister’s disease?

A: If (my brother or sister’s disease is) not cured (he/she) will die, and feels painful.

Q: But then why don’t you take the money to cure your own disease (in the above case)?

A: I would rather let myself suffer the pain. I don’t want to see my brother suffer the pain. If my brother and sister are sick, members of my family would be very sad.

Q: How about your disease?

A: If I am sick, I won’t let my family members know. I don’t want them feel worried.

The subject exhibits a deep concern for her family members’ sickness and worries. She would rather sacrifice herself for her family members. Her empathy toward her siblings and other family members appears to be deep.

##### Hierarchy of human relationships: kin, good friends, and strangers

The following Hierarchy of Human Relationships: kin, good friends, and strangers is emphasized here. In other words, the tendency to perform altruistic behavior toward others by people at this stage decreases consistently in the following order: kin, good friends, and strangers. This hierarchy of human relationships also reflects an important aspect of primary group altruism. People at this stage are more altruistic toward in-group members or someone closely related than toward out-group members or someone less related. The primary group here usually refers to one’s family or a gang of which one is member. A female undergraduate subject responded to “The Sinking Boat” as follows:

Q: Your decision to rescue your brother or sister is definitely yes, and to rescue your best friends is strongly yes. For other people, your decision is moderately yes or moderately no. Why?

A: Mainly because of the additional affection (for brother, sister, and best friends)….

Q: Do you think the act is selfish or natural?

A: I think it is very natural. Although the basic rights of all of us are the same, but there is an additional factor of affection for him (the brother, sister, or best friend). After all, if you have to choose between two types of people, who is your priority? It is natural to choose the one, I mean, with affection and relationship.

The above subject’s responses clearly reflect the above Hierarchy of Human Relationships in various situations.

#### Justice reasoning: mutual interpersonal expectations

People at this stage live up to what is expected by members of their primary group (e.g., family, school, religious, or political parties) or people close to them.

##### Meeting the group’s expectation

People at this stage live up to “what is expected by people close to you or what people generally expect of people in your role as son, brother, friend, etc.” ((93), p. 34). In other words, the right behaviors are those which can earn approval from the group. In short, it is a “good-boy-nice-girl orientation” [(14), p. 18].

One of Kohlberg’s (15) hypothetical dilemmas which is called “Joe and his father” is used here for illustrative purpose. The major content of this dilemma is as follows: Joe is a boy who wanted to go to camp very much. His father promised him he could go if he saved up the money for it himself. So, he worked hard at his paper route and saved enough money to go to camp. But then his father was short of money to go on a special fishing trip with his friends and told Joe to give him the money he had saved from the paper route.

A male first-year undergraduate responded to the “Joe and His Father” dilemma as follows:

Q: Should Joe refuse to give his father the money?

A: Yes. Because his father made it clear that Joe could go to camp if he could save enough money, and Joe worked hard to save the money. Since both parties are going for fun, his father has no reason to exercise his authority to order his son to give him the money.

Q: Does giving the money have anything to do with being a good son?

A: No. Since my parents have spent unaccountable amount of money and effort to nurture me, as a son or daughter, even though the money is earned by me, giving it to father is very appropriate. The most important thing is that his father has promised before that if Joe could save enough money, he could go to camp. It would be a different matter if (Joe’s) father has given him the money to go to camp and now (his father) wants to get it back.

The major concern in this subject’s response is that Joe has worked hard to earn the money. In addition, it is important that Joe’s father should keep his promise to let Joe go to camp since Joe has saved enough money himself. The above subject further elaborated his view on the role of a good son as follows:

Q: What do you think is the most important thing a son should be concerned about in his relationship to his father?

A: Filial piety. This includes love and affection toward parents, and also not letting parents suffer. For example if you earn $3,000, then you should give at least one-half of the money to support them. In addition, you should take time to accompany them and take care of them when they are sick.

Q: Providing (materialistic) support and caring to parents, which one is more important?

A: Caring. If you can earn several $10,000, you don’t need to give your parents several $10,000. But if you can only earn $3,000, then you need to give your parents one-half. Caring means try your best to let (your parents) enjoy their old age.

The subject’s major concern is to show caring and love toward parents. He tries to elaborate his view on the role of a good son. It is important to try one’s best to take care of one’s parents so that parents can enjoy their old age.

##### The authority of group leader

The rules governing the group members’ behavior are often made and administered by the group leaders, sometimes in consultation with the group members. What is right at this stage is thus to be loyal to the group, to trust, and respect the leaders, and to follow the rules set by the leaders. If conflict occurs, the leaders have final say and people at this stage would suppress or give up their own opinion and stick to the group’s rule or the leader’s decision. Group order is basically maintained by a style similar to parental control over children.

In the “Joe and his father” dilemma, a 21-year-old female undergraduate attempted to resolve the conflict between Joe and his father by filial piety.

Q: Is filial piety important?

A: Yes. Because filial piety in general refers to the respect and obedience to the superior by the junior. If the junior does not show respect and obedience to the superior, the superior might feel ignored or humiliated. If we live together in harmony, the conflicts (between Joe and his father) will not happen.

The emphasis with this subject is on respect and obedience to the superior or in general the group leader.

##### Primary group norms

When there is a conflict of interests between the primary group and an individual, people at this stage think that the rights, whether basic or relative, of the primary group should be protected at the expense of the individual. The norms of the primary group should be complied with by all means in all situations, and the interests of the group precede all the other things including social law, personal interests (e.g., self-actualization aspiration, or motivations), or moral principles such as universal justice and universal love. In the extreme case, the person regards himself or herself as a member of the group forever: To live as a man (woman) of the group, and to die as a ghost of the group too. One of the common norms of a primary group is that members of the primary group, in particular the young and junior ones, have the responsibility to contribute to the primary group in order to maintain its survival and prosperity. In other words, the survival of the primary group precedes that of an individual. A female undergraduate responded to “A Doctor’s Dilemma” as follows:

Q: Why would you give up the present job in the village if your mother suffers a stroke and wants you to come back?

A: Here I suppose that she can’t take care of herself and no one is available to take care of her. Since I am the only one that can take care of her, I therefore have to go back to look after her.

Q: That is, you feel that your (purpose for) return is to take care of her.

A: Yes.

Q: Why would you give up the job in the village to take care of your mother?

A: It is difficult to say, except that I feel I am the only one who can help members of the family. As for the job in the village which I give up, may be after I leave, there is still a chance that it will be carried out by someone else. But if I do not go back, there will be no chance for my mother to be taken care of by someone.

One of the primary group norms is the norm of filial piety which prescribes that people should take care of their parents at the expense of their own development and interests. The subject in the above case is willing to give up her job in the village which means that she is willing to give up her self-actualizing act to return to her country to take care of her mother.

### Differences between stages

For each of the parameters (psychological needs, altruism and human relationships, and justice reasoning), the differences in the developmental characteristics between stages are delineated in details.

#### Psychological needs

##### Physiological and safety needs

Stage 1 places greater emphasis predominantly on the physical survival needs, that is, physiological and safety needs. The person acts and functions more or less like a pet. Some psychologists [e.g., in Kegan’s (94) stage of incorporative self and in Loevinger’s (24) presocial stage of ego development] have constructed a “Stage 0” which deals with the most primitive and most biological and least socialized aspect of moral development. The major features of the Stage 0 are basically innate and the behavior is quite similar to that of a wild animal. Stage 1 children have been socialized and have developed some pre-operational cognition (95). While some of the animal characteristics (e.g., survival instinct and skills) still exist at this stage, they act like a pet rather than like a wild animal. For survival purposes and personal comfort, they act to obey their parents or other authorities. Sigmund Freud’s concepts of id and ego can also be applied here. The id is exclusively unconscious and consists of all the primitive inherited impulses, including the instincts of sex and aggression, present at birth. The id is said to operate in terms of the pleasure principles and functions in terms of the primary process. That is, the id attempts to avoid pain, reduce tension, and maximize pleasure or satisfaction irrationally. The ego develops from the id. The ego operates in terms of the reality principle in the sense that it attempts to take the real world into consideration in the process of the achievement of satisfaction. In other words, the ego tries to adapt to reality with the power of energy derived from the id (57, 96). The features of the Stage 0 tend to be similar to those of the id. On the other hand, Stage 1 is a starting stage of ego development but still possesses some features of id. In other words, the moral orientation at Stage 1 is based on the pleasure principle subject to the obedience to authority principle. As the person progresses to Stage 2 and then Stage 3, the emphasis is widened to include other basic needs. But the degree of pre-potency is still the highest for the physiological needs and decreases consistently to safety, belongingness, esteem, and self-actualization needs (38).

##### Belongingness and love needs

Love needs become a major emphasis at Stage 2 and belongingness needs are predominant at Stage 3. Stage 2 people focus on their self-happiness in terms of dominance and love needs. They are more concerned with their happiness, their interests, and their affection or love needs. The moral orientation at Stage 2 is based on the principle of opportunistic hedonism (24). People at this stage are usually self-protective, manipulative, exploitative, dominant, and opportunistic. In addition, Stage 2 people try to gratify their affection and love needs based on reciprocity and instrumental purposes. Stage 3 people focus more on belongingness needs. They place great emphasis on family love, family interests, and family cohesion. The identity of the primary group is clear. The interests and esteem or reputation of the primary group are their major concern at this stage.

##### Esteem needs

The esteem needs are less gratified at Stage 1 and gradually become a concern around Stage 3. At Stage 3, one’s esteem is tied with the esteem and identity of one’s primary group. The face, status, reputation, and dignity of the primary group are almost equivalent to one’s esteem or dignity. It is only after Stage 3 that one’s esteem is derived from an individualistic and autonomous self, independent of one’s primary group.

##### Self-actualization needs

In comparison to the other lower-order basic needs, the self-actualization needs are very much less emphasized or gratified at Stages 1 to 3. When there is a conflict between the self-actualization need and other basic needs, Stages 1 to 3 people tend to place more emphasis on lower-order needs.

##### Conflicts between two needs

When a moral decision involves a conflict between two needs, in comparison to lower stage people, higher stage people put more emphasis on higher-order needs. For example, when there is a conflict between physical survival (physiological and safety) needs and love needs, Stage 1 people place more emphasis on physical survival needs and less on love needs than the Stage 2 or 3 people. It should be remarked that as a person progresses to a higher stage, the variety of needs increases. However it does not mean that the lower basic needs will become totally unimportant or remain ungratified.

### Altruism and human relationships

Stage 1 empathy is more salient toward significant others such as parents. As people progress to Stage 2, they are able to play the role of others and understand others’ distress or suffering. They express more empathic distress toward their friends or intimates than toward strangers or other people. This is what Hoffman (10) calls “Friendship Bias” (p. 207). At Stage 3, this bias extends to members of primary group. In other words, people at Stage 3 express more empathic distress toward members of their primary group than toward out-group people. Hoffman (10) calls these biases the “Familiarity Bias” and “In-group Bias” (pp. 206–207).

#### Altruism

Stage 1 is a stage of egocentrism and authority-induced altruism. People at this stage are egocentric and selfish. They act altruistically only under the pressure or command of authority and for the sake of avoidance of physical punishment. On the other hand, Stage 2 people behave altruistically based on reciprocity and instrumental purposes. “You help me today and I will help you in future” is their reciprocity rule. When they progress to Stage 3, they act according to primary group altruism. That is, they act much more altruistically toward members of primary group than out-group people.

#### Human Relationships

Attachment at Stage 0 or Loevinger’s [(24), pp. 15–16] symbiotic stage involves the symbiotic relation with the mother. At this stage, children are not able to differentiate clearly self from non-self. As they progress to Stage 1, the symbiosis is very much weakened, but the attachment to mother, father, and care-takers is still deep and profound. Altruistic behavior is performed either to the very significant others such as parents or under the command of authorities. At Stage 2, the attachment extends to sibling, best friends, intimates, and lovers. The attachment is no longer bottom-up. It is more less a reciprocal and horizontal relationship. When people reach Stage 3, the attachment is extended not only to individuals such as closely related people and best friends or lovers but also to primary groups such as family, school, or political or religious parties/groups.

### Justice reasoning

#### Obedience

At Stage 1, people blindly obey the commands or instructions of authorities. They think that what is right is to follow the authorities’ command. The obedience to avoid physical punishment at Stage 1 is changed to obedience for the sake of getting benefits at Stage 2. People at Stage 2 would choose to obey a rule or another person’s command because it would help them to get what they want. Obedience is a means to get what one wants. While the authorities at Stage 1 are usually one’s parents or teachers, the authority at Stage 3 is the leader of the primary group. The strong sense of identity to and deep attachment to the primary group motivates one to follow closely the command of the group leader.

#### Instrumental purposes: cost and benefits

Instrumental purposes are a major feature of the Stage 2, but they also appear to some extent at other stages. At Stage 1, the instrumental purpose is in some sense quite egocentric. Things are regarded as right if they lead to win or gain. Whether the other person wins or gains is not the subject’s concern at this stage. In other words, the rule is that “I must win, whether you win or lose is not my concern”. Stage 2 people regard things as right for instrumental purpose if it leads to a win-win result, that is, “I win and you win too.” In other words, it should be a fair and beneficial deal for both parties. The Machiavellian feature of Stage 2 is a negative aspect of the instrumental purpose characteristic. The Machiavellian feature is something like “I must win any way even though you have to lose.” The instrumental purpose at Stage 3 focuses on interaction with the primary group. The golden rule is that “whether I would lose or win, I must act to make the primary group to win”.

The contents of cost and benefits tend to be concrete, materialistic, and quantitative at lower stages and gradually become abstract, spiritual, and qualitative at higher stages. In addition, the duration of the return of a social investment (48–51) is usually shorter at Stages 1 or 2 and longer at Stage 3 or above.

#### Norm-abiding

People at Stage 1 regard the social norms as rigid and inflexible. In addition, the rules set by each others are unchangeable at Stage 1. For Stage 2 people, norms, or rules are abided by for the sake of instrumental purposes. If norm-abiding or rule-abiding does not bring one benefits, then they are ignored. On the other hand, Stage 3 people strongly abide by the norms and rules agreed by their primary group but are much less strongly influenced by norms and rules set by people outside their primary group.

### A summary of the characteristics of the stage structures

#### Stage 1

At the first stage of moral development, the emphasis is on physical survival (physiological and safety needs), egoism and selfish orientation, and obedience to authorities. A deep and profound attachment to parents, empathy toward the significant others, obedience to authorities, and pet-like behavior all contribute to the physical survival of a person at this stage. For survival and an easy life, the subject has to live under the command and guidance of the authority. The subject’s emotion, thinking and behavior are totally controlled by the authority. In addition, an egocentric perspective and a selfish orientation indicate the narrow and limited concept of self at this stage.

#### Stage 2

Stage 2 people place emphasis on love needs, reciprocal altruism, opportunistic hedonism and instrumental purposes. People at this stage are self-protective, dominant, exploitative, and opportunistic. In order to protect one’s interests and in order to be in power or to be dominant, one can be Machiavellian and act according to the principle of opportunistic hedonism. On the other hand, reciprocal altruism and instrumental purpose serve as guiding principles in their affective and love interaction with others. In simple terms, it is a case of “you love me and I will love you too.” The need to love and to be loved is gratified on the basis of reciprocal altruism.

#### Stage 3

Stage 3 people place emphasis on belongingness needs, primary group altruism, and group identity and expectations. The central theme of this stage concerns the primary group. People at this stage have a strong desire to gratify their belongingness needs to a primary group. They are willing to sacrifice for the benefits of the group (e.g., group survival, group affection, group dignity, group interests, etc.) at great cost. They treat group members significantly differently from out-group members. They regard in-group members as siblings and friends and are willing to perform altruistic behavior toward them. They treat others as strangers and are much less altruistic to them in comparison to primary group members.

A summary of the characteristics of the three stages of moral development is presented in Table 1.

**Table 1:** **A summary of the stage characteristics**.

Stage	Characteristics		
	Psychological needs	Altruism and human relationships	Justice reasoning
1	Physical survival orientation: emphasis mainly on physiological and safety needs	Empathy: express more empathetic distress toward significant others such as parents	Obedience to authority: what is right is what the authorities say
		Selfish orientation: mainly taking care of one’s own interests	Egocentric viewpoint: only able to view matters from one’s role or perspective
	Pleasure principle: emphasis on seeking pleasure, happiness, and comfort	Human relationships: deep and profound attachment to parents	Rigid social norms and unchangeable rules: norms, proprieties, and usual practices must be followed as closely as possible; rules cannot be changed once agreed or set up except by authorities
		Authority-induced Altruism: to act altruistically following the commands or wishes of the parents or authorities	
2	Love needs: the need to love and to be loved	Empathy: express more empathic distress toward their lover and friends than toward strangers or other people (friendship bias)Altruism: reciprocal altruism	Instrumental purpose: acts are instrumental means to serve one’s needs and interest
	Need for companionship: the need for spouse, friends, and partners		Opportunistic hedonismTend to be Machiavellian in maintaining one’s survival and getting what one wantsNorm of equal exchange: compliance to social norms is based on instrumental purpose and equal exchange
		Friendship and peer relationships: affection extends to sibling, best friends, intimates, and lovers	
3	Belongingness needs: strong need to attach to a primary group (family, school, religious, or political party)	Empathy: express more empathic distress toward members of the primary group than toward external people (familiarity bias or in-group bias)	Meeting the group’s expectations: a good-boy-nice-girl orientation
	Group identity: esteem, dignity, reputation, and social status of the group	Primary group altruism: tend to be more altruistic to members of the primary group than to out-group members	Authority of group leader: to trust and respect the group leader and to follow the rules set up by the leader
		Hierarchy of human relationships: kin, good friends, and strangers	Primary group norms: the norm of the primary group should be complied by all means in all situations

## Concluding Remarks

In the construction of the first three stages of moral development, the author has employed a number of psychological theories. In particular, Maslow’s (38) Hierarchy of Basic Needs was used to elaborate the psychological needs aspect; Hoffman’s (10) theory of empathy and moral development, Loevinger’s (24) theory of ego development, and Sociobiological Theory (39, 52, 97) to explain the altruism and human relationships aspect; and finally Piaget (13) and Kohlberg’s (14, 15) theories to describe the justice reasoning aspect. The proposed theoretical model attempts to integrate the affective and cognitive aspects of moral development. It is hypothesized that the sequence of these three stages is invariant of person and culture. The present theory is based on the author’s previous work (90, 91, 98). The extension of this theory to stages above Stage 3 will be our next project. While the lower stages appear to be invariant of culture, the higher stages are hypothesized to be culture-bound. In addition, the present theory serves as an important basis for constructing the scoring manual of the stages of moral development for our future empirical work on moral intelligence and moral behavior.

In short, the present paper is a new attempt to divide the structure of moral development into three parameters: psychological needs, altruism and human relationships, and justice reasoning. It is a theory which attempts to integrate survival needs, love and belonging needs, esteem needs, self-actualization needs, altruism toward different kinds of people (kin, spouse, close relatives, best friends, acquaintance, strangers who are weak, young, old or elite of the society, common strangers, and enemies or someone you dislike), and justice reasoning into one single theoretical model.

This research was partially supported by research grants from Hong Kong Baptist University. Correspondence should be addressed to Hing Keung Ma, Department of Education Studies, Hong Kong Baptist University, Kowloon Tong, Hong Kong.

## Conflict of Interest Statement

The author declares that the research was conducted in the absence of any commercial or financial relationships that could be construed as a potential conflict of interest.
